# Chronic Heart Failure and Coronary Artery Disease: Pharmacological Treatment and Cardiac Rehabilitation

**DOI:** 10.3390/medicina61020211

**Published:** 2025-01-24

**Authors:** Maria-Alexandra Ciuca-Pană, Aristi Boulmpou, Cigdem Ileri, Giovanna Manzi, Michele Golino, Marina Ostojic, Akhmetzhan Galimzhanov, Stefan Busnatu, Simona Mega, Francesco Perone

**Affiliations:** 1 Cardiology Department, Carol Davila University of Medicine and Pharmacy, Bagdasar-Arseni Emergency Clinical Hospital, 041915 Bucharest, Romania; maria.alexandra.pana@drd.umfcd.ro (M.-A.C.-P.); stefan.busnatu@umfcd.ro (S.B.); 2 Third Department of Cardiology, Aristotle University of Thessaloniki, Ippokratio General Hospital, 54636 Thessaloniki, Greece; aristi_bou1993@yahoo.gr; 3 Cardiology Department, LIV Hospital Vadi Istanbul, Istanbul 34396, Turkey; cgdmileri@gmail.com; 4 Department of Clinical, Internal, Anesthesiology and Cardiovascular Sciences, Policlinico Universitario Umberto I, Sapienza University of Rome, 00161 Rome, Italy; giovanna.manzi@uniroma1.it; 5 Pauley Heart Center, Virginia Commonwealth University, Richmond, VA 23284, USA; micheleg1390@gmail.com; 6 Robert M. Berne Cardiovascular Research Center, Division of Cardiology, University of Virginia, Charlottesville, VA 22903, USA; 7 Cardiology Clinic, University Clinical Center of Serbia, 11000 Belgrade, Serbia; drmarinaostojic@gmail.com; 8 Department of the Propaedeutics of Internal Diseases, Semey Medical University, Semey 071400, Abai Region, Kazakhstan; ahmedgalimzhan@gmail.com; 9 Department of Cardiovascular Science, Campus Bio-Medico University of Rome, 00128 Rome, Italy; s.mega@policlinicocampus.it; 10 Cardiac Rehabilitation Unit, Rehabilitation Clinic “Villa delle Magnolie”, Castel Morrone, 81020 Caserta, Italy

**Keywords:** chronic heart failure, coronary artery disease, cardiac rehabilitation, guideline-directed medical therapy, pharmacological treatment

## Abstract

Coronary artery disease is the leading cause of acute and chronic heart failure. Patients with heart failure and ischemic heart disease need a tailored assessment to define the appropriate treatment, while a specific multidisciplinary management plan should be followed. Indeed, several factors should be assessed before starting treatment, such as heart failure symptoms and/or signs, angina, electrocardiographic features, right and left ventricular systolic and diastolic function, serological abnormalities, cardiac structural and functional integrity, and pulmonary function. New scenarios and developments have emerged recently in this field, increasing our knowledge regarding pathophysiology, exercise, and pharmacology. Effective and appropriate management and treatment reduce the risk of death and hospitalization for heart failure. Herein, we provide an updated, state-of-the-art overview of pharmacological treatment and cardiac rehabilitation in patients with chronic heart failure and coronary artery disease. Furthermore, tailored and contemporary management in clinical practice will be proposed for this specific and fragile patient population.

## 1. Introduction

**Figure 1:**
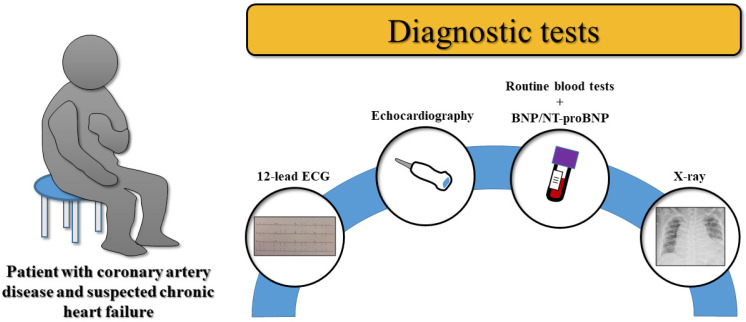
Diagnostic tests in patients with suspected chronic heart failure. The diagnostic algorithm for a patient with chronic coronary syndrome and suspected chronic heart failure is composed of a twelve-lead electrocardiogram, routine blood test, natriuretic peptide dosage, chest radiography, and transthoracic echocardiography. 12-lead ECG = twelve-lead electrocardiogram; BNP = B-type natriuretic peptide; NT-proBNP = N-terminal pro-B-type natriuretic peptide; X-ray = chest radiography.

Heart failure (HF) is a pluri-etiological syndrome with an alarmingly high incidence, which affects patients’ quality of life (QoL), causes frequent hospitalizations, and increases the risk of premature death. One of the most common etiologies of HF is ischemic heart disease (IHD), a pathology characterized by reduced myocardial blood flow resulting in relaxation and/or contraction dysfunction [1]. Since the 1970s, our pathophysiological knowledge about IHD has gradually increased. Via angiography techniques, it has been demonstrated that a reduction in coronary artery diameter by over 50% leads to vasodilator dysfunction, while a reduction of over 70% leads to low blood flow even at rest. Thus, the physio-pathological mechanism by which the narrowing of coronary arteries leads to myocardial ischemia and subsequent HF was established, with a survival rate inversely proportional to the severity of IHD. However, despite the central role that coronary atherosclerosis plays in IHD, current studies consider it only one component in a much longer and more complex process [2,3]. The 2024 European Society of Cardiology (ESC) Guidelines for the management of chronic coronary syndromes (CCSs) extends the etiology of myocardial ischemia to both macro- and microvascular compartments. Up to this point, researchers’ attention has been focused mainly on hemodynamically significant macrovascular lesions; therefore, the previous ESC guidelines did not consider hemodynamically insignificant lesions as having such a great impact on patients’ symptomatology and heart failure prognosis. But according to the new recommendations, fixed stenosis and diffuse atherosclerotic lesions can also cause ischemia under stress conditions, along with structural abnormalities (myocardial bridging, dynamic epicardial vasospasm, or congenital arterial anomalies). From a microvascular point of view, microcirculation coronary bed dysfunction is considered a predominant factor in the entire CCS spectrum, leading to the presence of angina-like symptoms even in the absence of large or medium obstructive coronary artery disease (CAD), thus resulting in the terms angina with non-obstructive coronary arteries (ANOCA) and ischemia with non-obstructive coronary arteries (INOCA) [4]. Typical HF symptoms include dyspnea at different degrees of exertion, decreased exercise tolerance, and ankle swelling, alongside the classic symptoms of IHD represented by strangling/constricting/squeezing retrosternal chest pain, extending to arm/jugular/interscapular regions, triggered by physical or emotional stress, relieved at rest or by sublingual nitroglycerin [5]. The diagnostic algorithm recommends practitioners to suspect HF in patients with cardiovascular risk factors, positive symptoms, and/or a modified at-rest electrocardiogram (ECG), and to proceed with natriuretic peptide collection and echocardiography to confirm HF and define its phenotype based on the left ventricular ejection fraction (LVEF) measurement (Figure 1). Based on the LVEF, HF can be categorized into HF with a preserved ejection fraction (HFpEF, LVEF ≥ 50%), mildly reduced ejection fraction (HFmrEF, LVEF 41–49%), or reduced ejection fraction (HFrEF, LVEF ≤ 40%). On the other hand, in patients without a precise etiological diagnosis, the risk estimation of obstructive epicardial CAD can be performed using the Risk Factor-weighted Clinical Likelihood model, adjusted according to the additional paraclinical investigations carried out or the patient’s medical history. This risk stratification test is newly introduced as a Class I recommendation in the 2024 ESC Guidelines for the management of chronic coronary syndromes and draws attention to the importance of a thorough clinical evaluation of the patient. Thus, depending on the risk of CAD, it is recommended that patients at low-to-moderate pre-test risk (>5–50%) continue myocardial ischemia evaluation using non-invasive techniques such as coronary computed tomography angiography or functional imaging, while in the case of patients at high risk (>50–85%), functional imaging assessment is suggested. Instead, in individuals with very high risk (>85%), invasive coronary angiography is recommended [4,5,6]. For moderate–high-risk patients, imaging evaluation through stress echocardiography, positron emission tomography, and cardiac magnetic resonance perfusion imaging, if available, received a Class I recommendation according to the new 2024 ESC Guidelines for the management of chronic coronary syndromes. Tailored assessment plays a key role in the diagnosis and management of patients with ischemic HF to ensure adequate treatment with minimum complications. Therefore, this review aims to highlight the new concepts considered in patients with ischemic HF and the importance of tailored assessment in adequately prescribing pharmacological and non-pharmacological treatment and multidisciplinary management.

## 2. Multidisciplinary Management and Cardiac Rehabilitation

**Figure 2:**
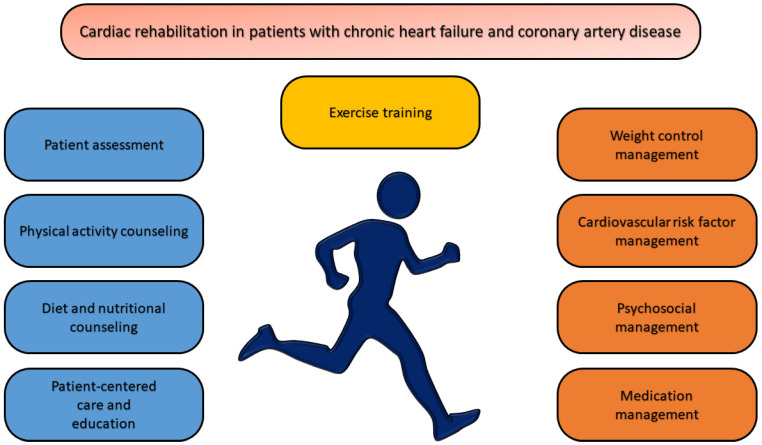
Core components of cardiac rehabilitation program in patients with chronic heart failure and coronary artery disease. The cardiac rehabilitation program for patients with chronic heart failure and coronary artery disease has the following components: patient assessment, physical activity counseling, diet and nutritional counseling, patient-centered care and education, exercise training, weight control management, cardiovascular risk factor management, psychosocial management, and medication management.

Since the 2016 ESC Guidelines for the diagnosis and treatment of acute and chronic HF were published, a multidisciplinary approach to patients with HF has been recommended, including evidence-based diagnosis, adequate therapy, medical education, and appropriate follow-up. A multidisciplinary patient-focused HF management program (HF-MP) includes disease prevention, symptom control, and the palliative management of patients with end-stage HF, with professional medical staff that encourages patients’ adherence to the prescribed treatment. Moreover, an HF-MP should monitor and correct patients’ lifestyle and pharmacological treatment, assess implantable device status, provide psychosocial support, diagnose and manage acute decompensation, and ensure patient access to advanced therapies [7]. There are several care models for patients with ischemic HF, from clinic-based to home-based and even hybrid models. In a meta-analysis published in 2017 that included 53 randomized clinical studies, both HF-MPs on-site and at patients’ homes reduced all-cause mortality compared to the usual model of care. Although both models of care were effective, those at the patients’ homes were superior regarding positive outcomes [8]. The technological devices used in implementing such secondary prevention services vary greatly, with no specific differences in effectiveness. The positive features of HF-MPs, with better results in terms of morbidity and mortality, are the holistic approach to all cardiovascular risk factors and comorbidities, adaptation to the available infrastructure and resources, and the customization of administration policies according to patients’ necessities [9,10]. HF-MPs can be organized through cardiovascular rehabilitation programs, given that many of the recommended components overlap with the components of a classic cardiac rehabilitation (CR) program. Maybe the solution to the adequate management of ischemic HF could be a proper enrolment of patients in cardiovascular rehabilitation programs. The following HF-MP-recommended components can be achieved through a CR program: medical education for self-care, physical activity and exercise, nutrition, withdrawal from tobacco, alcohol, or recreational drugs, symptom monitoring and self-management, and anxiety and depression management [11]. The specialized literature draws attention to the importance of patients’ medical education, demonstrating that patients with increased self-care have a better QoL, lower hospitalization rates, and lower mortality. Emphasis is also placed on individualized medical education according to the educational and psychological background and patients’ comorbidities by offering various formats and encouraging active roles for patients and their caregivers [12]. Participation in CR programs for patients with ischemic HF, especially those with HFrEF, is recommended to start as soon as possible after diagnosis and/or the acute event to ensure patients’ social reintegration and to avoid short-term complications, the progression of atherosclerotic disease, and all-cause mortality. Physical exercise prescription for patients with ischemic HF should follow the FITT (frequency, intensity, time duration, and type of exercise) model [13,14] (Figure 2). However, although exercise is essential in the management of ischemic HF disease, the participation rates are extremely low among patients. Evidence from many clinical trials and meta-analyses describes improved functional capacity, peak oxygen consumption (VO_2_ max), and QoL in patients with HFrEF enrolled in CR programs. Furthermore, in highly adherent patients, hospitalization rates are lower than in those who do not participate in rehabilitation programs. In recent years, the implementation of remote CR programs with or without telemonitoring has increased, and patient adherence to remote CR programs appears to be better. Perhaps the delivery of CR via technology-based approaches will support patients’ adherence to a healthier lifestyle and the more effective secondary prevention of chronic HF and CAD [15].

## 3. Pharmacological Treatments for Patients with HFpEF and CAD

After the inconclusive results of many large-scale studies in patients with HFpEF, for a long time, the only guideline recommendation for HFpEF treatment was to reduce congestion and control comorbidities [5]. However, sodium–glucose cotransporter 2 (SGLT2) inhibitors are now the only recommended option to modify the disease progression of HFpEF. In the DELIVER study, dapagliflozin reduced all-cause mortality and HF hospitalization and provided symptomatic and physical improvement in patients with HFpEF [16,17]. In the EMPEROR-Preserved study, empagliflozin significantly benefited the primary composite outcome [18]. Considering these positive large-scale studies, the 2023 Focused Update of the ESC HF Guidelines recommend SGLT2 inhibitors as a Class I indication in treating HFpEF [19]. Instead, despite well-conducted studies on the targeted use of the angiotensin receptor–neprilysin inhibitor (ARNI) and mineralocorticoid receptor antagonist (MRA) in HFpEF, current ESC guidelines do not endorse their use due to a lack of definitive evidence from clinical research [19]. However, the American College of Cardiology (ACC) guidelines recommend the use of ARNI and MRA [20]. More than half of patients with HFpEF have accompanying obstructive CAD, and concomitant CAD worsens the prognosis [21]. Among patients without obstructive CAD, 81% had coronary microvascular dysfunction [22]. Therefore, antianginal treatment and recommendations that may be required in addition to HF treatment in these patients are necessary and fundamental for long-term management. Beta-blockers control heart rate by decreasing sympathetic stimulation and reducing peripheral vascular resistance. In the ELANDD study, although heart rate was controlled with 6 months of beta-blocker therapy, no improvement in exercise capacity was observed in HFpEF [23]. Currently, the advantages of beta-blockers are still in question. A recent meta-analysis based on observational studies showed that beta-blocker therapy reduced all-cause mortality in patients with HFpEF but did not affect rehospitalization for HF [24]. Another meta-analysis concluded that beta-blocker treatment reduced cardiovascular mortality but did not change all-cause mortality and HF hospitalization [25]. The recent DELIVER study showed that using beta-blockers did not increase the risk of HF hospitalization and all-cause mortality [26]. In addition to the lack of agreement on their benefits, there are also concerns that beta-blockers might adversely impact clinical results through chronotropic incompetence. Routine use of beta-blockers in HFpEF is not recommended in the ESC and AHA/ACC/HFSA HF guidelines, but their use in the presence of CAD and angina is supported [27]. Non-dihydropyridine calcium channel blockers (CCBs) are often poorly tolerated in HF due to their negative inotropic properties. Conversely, dihydropyridine CCBs tend to have a low impact on myocardial contractility and may effectively decrease left ventricular afterload [20]. According to a recent meta-analysis, regardless of the subgroup, using CCBs in HFpEF does not affect prognosis [28]. Findings across many randomized controlled trials (RCTs) and cohort studies investigating the effects of CCBs on exercise capacity in HFpEF are inconsistent [29,30]. CCBs may be utilized to manage angina in HFpEF, as they have not been shown to cause harm, but are contraindicated in HFrEF [20]. The ESC HF guidelines suggest using CCBs for managing heart rate and alleviating angina, although they do not offer direct advantages for HF management or coronary outcomes [5]. In the NEAT-HFpEF study, nitrate use did not improve QoL scores or 6-min walk distances and reduced daily activity levels in patients with symptomatic HFpEF [31]. Ivabradine does not affect HF hospitalization and all-cause mortality, and data on left ventricular diastolic function and exercise capacity remain controversial in HFpEF [32,33]. In the RALI DHF study, ranolazine did not improve echocardiographic relaxation parameters [34]. In the DOPING-HFpEF study, trimetazidine did not improve diastolic function and myocardial energy homeostasis in HFpEF [35]. In light of these data, the use of these interventions for angina relief in patients with HFpEF has been recommended by the 2024 ESC guidelines, since their benefits on the outcome of HF have not been demonstrated [4] (Table 1).

**Table 1:** Major randomized controlled trials involving patients with EF ≥ 50%.

Study	Intervention	Left Ventricular EF
DELIVER [16]	Dapagliflozin	>40%
DETERMINE-PRESERVED [36]	Dapagliflozin	>40%
EMPEROR-Preserved [18]	Empagliflozin	>40%
EMPERIAL-PRESERVED [37]	Empagliflozin	>40%
CHARM-Preserved [38]	Candesartan	>40%
I-PRESERVED [39]	Irbesartan	≥45%
PEACE [40]	Trandolapril	>40%
PEP-CHF [41]	Perindopril	>40%
TOPCAT [42]	Spironolactone	≥45%
SPIRRIT-HFpEF [43]	Spironolactone	≥40%
ALDO-DHF [44]	Spironolactone	>50%
PARAGON-HF [45]	Sacubitril–valsartan	≥45%
PARAGLIDE-HF [46]	Sacubitril–valsartan	>40%
SENIORS [47]	Nebivolol	>35%
J-DHF [48]	Carvedilol	>40%
ELANDD [23]	Nebivolol	>45%
NEAT-HFpEF [31]	Nitrate	≥50%
RALI DHF [34]	Ranolazine	≥45%
DOPING-HFpEF [35]	Trimetazidine	≥50%

EF—ejection fraction; HF—heart failure; HFpEF—heart failure with preserved ejection fraction.

## 4. Pharmacological Treatments for Patients with HFmrEF and CAD

Specific studies were not designed for HFmrEF, and the benefits from neurohormonal blockade therapy, such as using angiotensin-converting enzyme (ACE) inhibitors, the angiotensin receptor blocker (ARB), ARNI, MRA, and beta-blockers, were similar to those for HFrEF [49]. SOLOIST-WHF is the first study to support the use of SGLT2 inhibitors in treating HFmrEF or HFpEF. This study assessed sotagliflozin, which reduced cardiac outcomes in patients with HF and type 2 diabetes mellitus, regardless of LVEF [50], and inspired the EMPEROR-Preserved and DELIVER studies. In addition, in a recent study, treatment with SGLT2 inhibitors in HFmrEF was associated with improved biventricular function and ventricular dimensions at follow-up [51]. A meta-analysis of six studies concluded that SGLT2 inhibitors should be considered the primary treatment for all patients with HFmrEF [52]. Regarding drugs that act on the renin–angiotensin–aldosterone system, in the PEP-CHF trial, which included >70-year-old patients with an LVEF > 40%, perindopril showed symptomatic benefits, improved functional capacity, and reduced hospitalizations due to HF [41]. In the PEACE study, which enrolled patients with CCSs and a normal or mildly reduced LVEF, trandolapril had a favorable effect on MACE but did not reduce cardiovascular mortality and HF hospitalization [40]. However, in the I-PRESERVE study with a median follow-up of 4 years, irbesartan had no benefit in patients with LVEF ≥ 45% [39]. In the CHARM-Preserved trial, candesartan treatment reduced cardiovascular outcomes in patients with HFrEF and HFmrEF (LVEF < 50%) [38]. The landmark PARAGON-HF, which included patients with HF with LVEF ≥ 45%, showed no significant benefit of sacubitril–valsartan on cardiovascular outcomes [45]. However, in a subgroup analysis, sacubitril–valsartan was effective in patients with LVEF ≤ 57%. Pooled analysis data of PARAGLIDE-HF and PARAGON-HF support the use of sacubitril–valsartan in reducing cardiovascular events in patients with HFmrEF and HFpEF [53]. Finally, in the spline analysis of the TOPCAT trial, spironolactone in patients with HF with LVEF ≥ 45% showed benefits in the primary endpoints when the LVEF was <55% [42]. Due to the suggestive but inconclusive results of the TOPCAT trial, the SPIRIT-HF and SPIRRIT-HFpEF studies designed for MRAs (including eplerenone) are ongoing. Instead, in a meta-analysis of 11 studies, the use of beta-blocker therapy in HFmrEF with sinus rhythm had a positive effect on prognosis [54]. Likewise, in a large cohort of patients with LVEF ≥ 40%, beta-blocker use was potentially beneficial in patients with HFmrEF [55]. Data from the Swedish HF cohort suggested the mortality benefit of beta-blockers in patients with HFmrEF with CAD but not in HFmrEF without CAD [56]. Ivabradine, instead, is not recommended in HFmrEF as it did not improve cardiac endpoints in the EDIFY study [57,58] (Table 2).

**Table 2:** Major randomized controlled trials involving patients with EF 41–49%.

Study	Intervention	Left Ventricular EF
SOLOIST-WHF [50]	Sotagliflozin	<50%
DELIVER [16]	Dapagliflozin	>40%
DETERMINE-PRESERVED [36]	Dapagliflozin	>40%
EMPEROR-Preserved [18]	Empagliflozin	>40%
EMPERIAL-PRESERVED [37]	Empagliflozin	>40%
CHARM-Preserved [38]	Candesartan	>40%
I-PRESERVED [39]	Irbesartan	≥45%
PEP-CHF [41]	Perindopril	>40%
PEACE [40]	Trandolapril	>40%
TOPCAT [42]	Spironolactone	≥45%
SPIRRIT-HFpEF [43]	Spironolactone	≥40%
PARAGON-HF [45]	Sacubitril– valsartan	≥45%
PARAGLIDE-HF [46]	Sacubitril– valsartan	>40%
SENIORS [47]	Nebivolol	>35%
J-DHF [48]	Carvedilol	>40%
ELANDD [23]	Nebivolol	>45%
NEAT-HFpEF [31]	Nitrate	≥50%
RALI DHF [34]	Ranolazine	≥45%
DOPING-HFpEF [35]	Trimetazidine	≥50%

EF—ejection fraction; HF—heart failure; HFpEF—heart failure with preserved ejection fraction.

## 5. Pharmacological Treatments for Patients with HFrEF and CAD

CAD represents one of the leading causes of HFrEF, significantly contributing to increasing morbidity and mortality in the Western world [59]. Advances in diagnostic and therapeutic strategies have markedly improved the care of patients with CAD, enhancing survival rates [60]. CAD remains a primary risk factor for the development of HFrEF [61]. In this context, pharmacological therapy is the cornerstone of the treatment of CAD-induced HF; over the last few decades, ground-breaking clinical trials have deepened our understanding of HF mechanisms, leading to substantial improvements in pharmacological treatments [62]. Historically, drugs such as ACE inhibitors, beta-blockers, and diuretics have been the main choices regarding the optimal management of HF [63]. More recently, treatment has further evolved to focus on the “four pillars” of HF care, with SGLT2 inhibitors being the latest addition to the HF treatment armamentarium [5,64]. For patients with HFrEF and CAD, pharmaceutical agents that positively impact ventricular remodeling and address key risk factors, such as arterial hypertension, have been proven to reduce morbidity, mortality, and HF hospitalizations while effectively relieving symptoms [65] (Table 3).

### 5.1. ACE Inhibitors/ARBs

As the renin–angiotensin–aldosterone system is fundamental to HF hemodynamics, ACE inhibitors are important in HFrEF medical management [66,67]. Large-scale clinical trials, such as the CONSENSUS and SOLVD trials, have proven the effectiveness of ACE inhibitors in reducing mortality and relieving symptoms and congestion [68,69]. By improving endothelial function and mitigating the progress of atherosclerosis, these agents have also proven beneficial in CAD and HFrEF [70]. At the same time, ARBs, acting in the same system but with fewer adverse effects, have not been proven to reduce mortality. Still, they relieve symptoms and reduce HF hospitalizations, with higher adherence rates [71]. ACE inhibitors and ARBs improve outcomes in patients with CAD primarily by inhibiting adverse myocardial remodeling, slowing the progression of ventricular dilation, and lowering the risk of HF; this reduces the incidence of hospitalization, myocardial infarction, and overall mortality [72,73].

### 5.2. ARNIs

The introduction of ARNIs into clinical practice, primarily supported by the results of the PARADIGM-HF trial, has revolutionized the treatment of patients with HFrEF [74]. Heart rate and myocardial oxygen demand are effectively reduced through the combined effect of ARB and neprilysin inhibition, which enhances natriuretic peptides and improves hemodynamics [75]. The exact role of ARNIs in stable CAD is yet to be determined, though there is emerging evidence that ARNIs may improve endothelial function and reduce arterial stiffness [76]. These benefits suggest that these agents may offer protection to vasculature, which could be particularly advantageous in patients with HFrEF and CAD.

### 5.3. Beta-Blockers

Beta-blockers have long been the cornerstone of the treatment of both HFrEF and CAD [77]. These agents act by antagonizing the effects of the sympathetic nervous system, specifically by blocking beta-adrenergic receptors, which reduces heart rate, myocardial contractility, and arterial blood pressure [78]. These effects are crucial in patients with HFrEF of ischemic etiology, leading to improved cardiac output [79]. In patients with HFrEF, beta-blockers reduce symptoms and improve long-term outcomes, including a significant reduction in HF hospitalizations and mortality [77,80]. Clinical trials such as MERIT-HF and CIBIS-II have demonstrated these benefits, particularly for metoprolol, carvedilol, and bisoprolol [81,82]. While data regarding the long-term effects of beta-blocker administration in patients with CAD without HF remain controversial, these agents play a pivotal role in patients with HFrEF by reducing the risk of arrhythmias and exerting benefits on ventricular remodeling [83]. In the same line, beta-blockers are proven to enhance survival in patients following myocardial infarction, especially when HF symptoms are present [84].

### 5.4. MRAs

MRAs, such as spironolactone and eplerenone, are crucial in managing HFrEF by blocking aldosterone receptors, reducing fluid retention, and limiting myocardial fibrosis [85]. Aldosterone contributes to vascular disease and is independently linked to increased mortality and ischemic events in patients with CAD [86]. In HFrEF, MRAs block the final component of the renin–angiotensin–aldosterone system, preventing harmful cardiac remodeling and fluid overload and eventually improving cardiac hemodynamics [85]. This leads to significant improvements in morbidity and mortality, as demonstrated in trials such as RALES and EMPHASIS-HF [87,88]. As one of the “four pillars” of HFrEF treatment, MRAs aid in reducing hospitalizations and improving survival [5].

### 5.5. SGLT2 Inhibitors

Initially developed for diabetes management, SGLT2 inhibitors have emerged as a novel and effective HFrEF therapy regardless of diabetes status [5]. These agents reduce glucose reabsorption in the proximal tubular epithelial cells of the kidneys, leading to mild diuresis, reduced arterial blood pressure, and decreased cardiac preload and afterload [89]. SGLT2 inhibitors have been demonstrated to significantly reduce the risk of HF hospitalizations and cardiovascular death in patients with HFrEF, as indicated in the DAPA-HF and EMPEROR-Reduced trials [90,91]. Their ability to improve HF outcomes and their renal-protective effects have enhanced their position as one of the cornerstones of HFrEF treatment [19,92]. In the same vein, patients with type 2 diabetes mellitus and extensive CAD seem to equally benefit from treatment with SGLT2 inhibitors, as it was associated with a reduced risk of all-cause mortality [93].

### 5.6. Antianginal Medication in Patients with CAD and HFrEF

The management of angina in patients with both CAD and HFrEF can be challenging, requiring a comprehensive approach to avoid worsening HF symptoms. Nitrates are commonly used to relieve angina by promoting vasodilation and reducing myocardial oxygen demand [4]. However, they must be used cautiously, as excessive vasodilation can lead to hypotension, which may be crucial in HFrEF [94]. Ranolazine is another agent available for the treatment of angina, particularly for patients with refractory symptoms, as it improves myocardial efficiency without significantly affecting heart rate or blood pressure [93,95]. CCBs, such as amlodipine, can be used selectively in patients with HFrEF to control angina. Still, non-dihydropyridine CCBs (e.g., verapamil, diltiazem) should be avoided due to their negative inotropic effects [96,97]. Overall, antianginal treatment in this special patient population must be tailored to balance ischemia relief with HF management.

## 6. Pharmacological Treatments for Patients with Right Ventricular HF and CAD

Right ventricular (RV) HF can complicate the clinical course of patients with myocardial infarction (MI) more acutely rather than as a late sequela. Indeed, in contrast to the left ventricle (LV), the RV has unique behavior in response to myocardial ischemia: RV dysfunction is more commonly mediated by the ischemic stunning of viable myocardium rather than irreversible myocardial necrosis, which explains its greater likelihood of recovery compared with the LV. The larger coronary oxygen reserve, the perfusion occurring throughout the cardiac cycle in systole and diastole due to the lower contractile pressures, and the collateral flow provided from the left anterior descending artery through the moderator band artery are all features that make the RV less susceptible to infarction than the LV [98]. The function of the RV is influenced by preload, afterload, and contractility. Therefore, beyond urgent revascularization, which is the cornerstone of the acute management of HF after RVMI, physicians should have the following goals when treating patients with RVHF [99]: 1. preload optimization; 2. afterload reduction; and 3. RV contractility enhancement.

1. A higher preload is needed to maintain forward flow when the RV output is reduced due to contractile dysfunction with a normal afterload (as in acute RV infarction). Therefore, patients with hypotension, without pulmonary congestion and with an estimated central venous pressure (CVP) < 15 mmHg and/or a normal pulmonary capillary wedge pressure (PCWP) should receive an intravenous fluid challenge with a bolus of 250 milliliters of normal saline solution. Careful hemodynamic monitoring is necessary to avoid excessive volume loading: an increase in CVP or PCWP of, respectively, >12 mmHg and >15 mmHg, without a parallel increase in blood pressure, should prompt the interruption of volume resuscitation. In this setting, the RV overload may be detrimental; the increase in RV dilation and a leftward shift of the interventricular septum may reduce LV filling and ultimately compromise cardiac output, precipitating a low-output state. Considering that patients with RV infarction are preload-dependent, the use of loop diuretics should require caution and should be reserved for patients with elevated CVP and signs of congestion. Similarly, treatments reducing RV preload, such as nitrates, opiates, and ventilation, should be avoided.

2. In patients with RVHF after MI, the use of pulmonary arterial vasodilators, currently approved for the treatment of pulmonary arterial hypertension (PAH), is not recommended, mainly because of their potential to induce systemic hypotension. To reduce RV afterload in patients with RVHF, clinicians should instead correct any conditions increasing pulmonary vascular resistance, such as hypoxia, acidosis, and hypercapnia.

3. The use of vasopressors and inotropes should be limited to patients with end-organ hypoperfusion and hemodynamic instability because of their potential for increasing oxygen consumption and arrhythmias [100]. Norepinephrine, which causes vasoconstriction through alpha-1 receptor stimulation, is the vasopressor of choice: it restores arterial blood pressures and improves organ and coronary perfusion without affecting pulmonary vascular resistance. In patients with RVHF and cardiogenic shock despite vasopressor infusion, inotropes are recommended. Dobutamine is the first-line inotrope in primary RV dysfunction; it resulted in improved hemodynamics (increase in cardiac index and stroke volume index) compared to volume loading alone [101].

When the listed therapeutic strategies are not sufficient to solve cardiogenic shock, mechanical support devices (MCS) should be considered as a “bridge to recovery” of RV function. Current options include venoarterial-extracorporeal membrane oxygenation (VA-ECMO) or dedicated systems such as the Impella-RP or the TandemHeart. Hemodynamic, clinical, laboratory, and imaging variables should guide clinicians in the decision to taper treatment or wean patients off MCS.

As in acute RHF, the management of patients with chronic RHF follows the same three principles listed above. In the chronic setting, the focus is on volume removal; high doses of diuretics, particularly a combination of loop diuretics and thiazides, are usually needed to maintain the delicate balance between relief from RV volume overload and congestion and sufficient preload to ensure adequate cardiac filling. In contrast to the clear guidelines for the management of HFrEF, advocating the quadruple combination therapy, evidence of disease-modifying treatment in patients with chronic right-sided HF after MI is poor. Recently, some experiments have been reported. Galves R. and colleagues [102] investigated the effects of beta-blockers on RV function in 40 patients with HF hospitalized within the preceding 12 months. Beta-blockers significantly improved the echo parameters of LV and RV remodeling. Masarone et al. [103] demonstrated the effect of sacubitril–valsartan in increasing tricuspid annular plane systolic excursion (TAPSE) and in reducing PAP with a consequent improvement in RV–pulmonary artery (PA) coupling, independent of LV remodeling, in 163 patients with HFrEF. Similarly, Yang and colleagues [104] showed that sacubitril–valsartan significantly improved RV function indicators (TAPSE, tricuspid annular s’ peak velocity, RV fractional area change, and pulmonary artery systolic pressure) in a single-center, retrospective observational study with 82 patients with HFrEF enrolled, who were followed for 6 months. An improvement in TAPSE and RV fractional area change (FAC) values compared to baseline has recently been observed, as well as the use of SGLT2 inhibitors, in an interesting meta-analysis, including eight studies with 370 patients with HF [105]. However, to our knowledge, no study has specifically evaluated the four pillars in a cohort of patients with RVHF after MI. Future studies are welcome to test their safety and efficacy in patients with RV dysfunction after MI, though mainly in an isolated manner, to exclude the possibility any improvement in RV ejection fraction is secondary to improved LV systolic function.

## 7. Tailored Management in Clinical Practice

**Figure 3:**
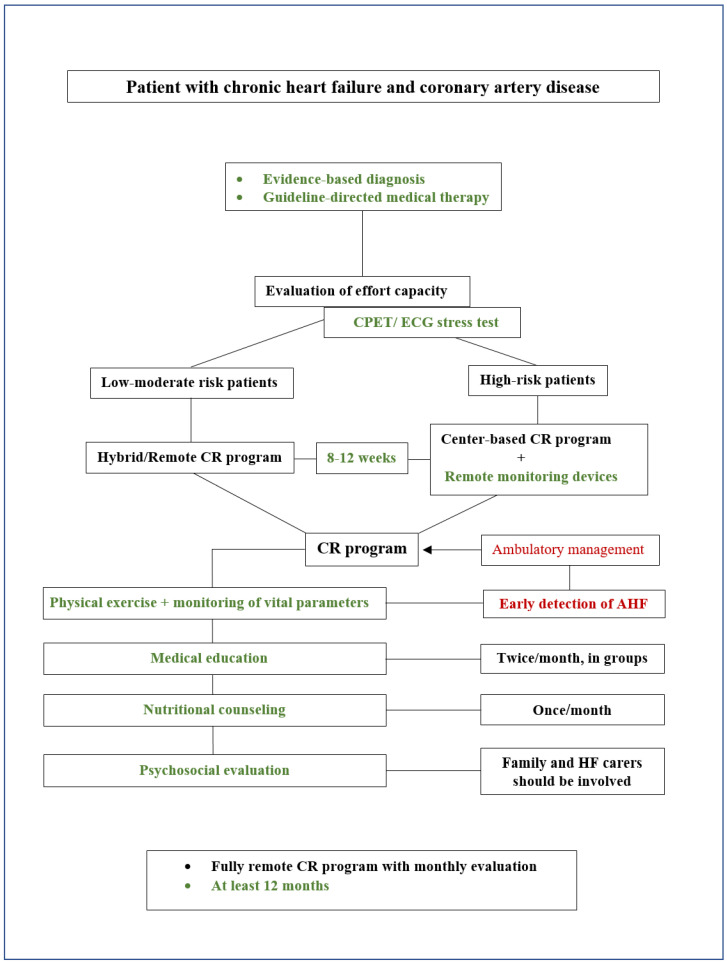
Management algorithm for patients with chronic heart failure and coronary artery disease: exercise assessment and prescription and cardiac rehabilitation program. AHF—acute heart failure; CPET—cardiopulmonary exercise test; CR—cardiac rehabilitation, ECG—electrocardiogram.

After the diagnosis and evidence-based treatment of chronic HF of ischemic etiology, patients should be referred to long-term HF management programs. To be effective, these types of programs should be cost-effective, be feasible for the medical infrastructure of the country where they are implemented, and offer all the monitoring components recommended for a patient with ischemic HF. Although pharmacological and interventional therapy for HF and CAD has evolved greatly in recent decades, alongside its patients’ life expectancy, its morbidity and mortality are increasing [106]. Recently, it has been observed that patients with HF do not receive adequate follow-up post-discharge, despite guideline recommendations, most probably due to the impossibility of following such a large number of patients without dedicated infrastructure and with few specific healthcare professionals. Thus, despite the adequate treatment that patients with HF and CAD receive during hospitalization, intervention in their lifestyle and social reintegration remains insufficient, and cardiovascular risk factors that contributed to the appearance of the underlying disease are poorly and inadequately treated. All these deficiencies in the long-term management of patients with ischemic HF lead to disease progression and the early appearance of complications. Considering that the recommended components of an HF-MP overlap with the components of a CR program, we propose integrating such a program into CR. This situation could be beneficial for the adequate management of patients with HF-CAD and for increasing enrolment in CR programs. It is important to mention that although the recommendations for the enrolment of patients with CAD in CR programs date back almost 40 years, the referral and effective enrolment of these patients remain extremely low [107]. Therefore, we propose a management algorithm for these patients, integrating both the HF management and cardiovascular prevention components, the importance of personalized assessment, and a more cost-effective approach without compromising the patient’s well-being (Figure 3). The algorithm proposes that immediately after a patient is diagnosed with chronic ischemic HF and has received the appropriate pharmacological therapy, they should undergo a functional capacity and cardiovascular risk assessment using an ECG stress test (preferably cardiopulmonary exercise testing). Depending on the ECG stress test results, patients should be divided into two groups: individuals at low–moderate risk or individuals at high risk of developing cardiovascular complications during physical exertion. Moderate–low risk patients will be further enrolled in hybrid CR programs or fully remote CR, while high-risk patients will be referred to center-based CR programs. All patients should have their vital parameters (such as heart rate, blood pressure, and oxygen blood saturation) monitored during the physical exercise sessions and outside of them to ensure the early detection of possible acute HF decompensation. Using a digital platform, these data could be stored automatically and available to the medical team at any time so that a patient who shows signs of HF decompensation can be sent to the outpatient clinic for evaluation and treatment optimization. Patients’ medical education is the key element of such a program, with the aim of fully understanding HF, CAD, possible complications, long-term prognosis, and the importance of adherence to pharmacological and non-pharmacological treatment. In addition, during the medical education sessions, cardiovascular risk factor management and adequate control and impact on the chronic HF condition should be addressed. Emphasis should also be placed on the importance of sleep, stress reduction techniques, and individualized levels of physical exertion that can be performed in daily life. All these elements promote an increased QoL for patients with HF. The medical education component should be carried out remotely, twice a month, and in groups to stimulate belonging, reduce the anxiety of patient–medical interaction, and stimulate role play [5]. Nutrition is a very important aspect of the balance of a patient with HF, having a key role in its impact on cardiovascular risk factors (such as weight, blood pressure, and diabetes mellitus) and its interaction with certain pharmacological therapies. This component should also occur remotely once a month and should be led by a nutrition specialist, who should provide food information and examples of individualized meals for each patient. Of all the components of a CR program, psychosocial assessment is probably the most absent, despite multiple studies showing the impact of inadequately treated anxiety and depression on the progression of cardiovascular disease. All patients with chronic HF should have a psychosocial assessment immediately after diagnosis, with continued therapy depending on each patient’s outcome. Both CR program models should have a recommended duration of 8–12 weeks, with the remote modality continued for a minimum period of 12 months and characterized by a reduction in the frequency of interventions to ensure adequate adoption of the new and beneficial lifestyle. Finally, a successful treatment strategy goes beyond pharmacological and interventional therapy, addressing a wide range of social and environmental determinants of health that significantly impact patient outcomes. Social determinants such as racial disparities, socioeconomic status, education, access to healthcare, and social support have been shown to influence HF prognosis, adherence to treatment, and overall QoL [108,109,110,111]. Addressing these factors through tailored interventions, social support programs, and community-based strategies can lead to better engagement in rehabilitation programs and improved long-term health outcomes [112,113]. Environmental factors, including air pollution, temperature variations, urban planning, and walkability, significantly affect HF management and patient recovery [114]. Exposure to high levels of air pollution has been linked to increased cardiovascular morbidity, while temperature extremes can exacerbate HF symptoms [115,116]. Urban design that promotes walkability and access to green spaces can encourage physical activity, which is vital for rehabilitation [117]. An interdisciplinary approach that integrates cardiologists, primary care physicians, rehabilitation specialists, social workers, and urban planners can thus optimize patient-centered care, addressing clinical needs and the broader context of health and well-being [118]. Furthermore, the integration of big data analytics into the interdisciplinary approach offers transformative potential in managing ischemic HF. By aggregating and analyzing large datasets that include clinical, environmental, and social determinants, healthcare providers can better understand the complex interactions between these factors and HF outcomes. Big data can facilitate the development of predictive models for HF exacerbations, enabling personalized interventions and the early detection of decompensation. Machine learning algorithms can identify patterns in patient data that may not be apparent through traditional analysis, improving the accuracy of risk stratification and treatment plans. Real-time data from wearable devices and digital health platforms also allow for continuous monitoring, providing insights into patient behavior, lifestyle, and response to therapy. This data-driven approach can guide individualized rehabilitation strategies, optimize resource allocation, and ultimately improve patient outcomes by addressing the multifaceted nature of HF management in the context of social and environmental factors [119,120].

The prognosis of patients with HF-CAD depends on and should be evaluated according to the patients’ clinical condition, natriuretic peptide levels, LVEF, the severity of the underlying atherosclerotic disease, and the impact of the low cardiac output on other organs and systems. In clinical practice, NYHA classification and the LVEF parameters are used to evaluate the prognosis of patients. This is why the long-term prognosis of a patient is often underestimated or overestimated, due to the lack of a holistic approach and the lack of objective parameters [121].

In patients with CAD and HF, pharmacological treatment should be individualized based on different phenotypes and to improve outcomes in accordance with published studies. Specifically, in HFpEF and CAD, studies like DELIVER and EMPEROR-Preserved have shown that SGLT2 inhibitors reduced mortality and heart failure hospitalizations and improved symptoms, leading to their strong recommendation for HFpEF in 2023 ESC guidelines. Despite promising studies, ARNI and MRA are not yet endorsed by the ESC due to insufficient clinical evidence, though the ACC supports their use. Many patients with HFpEF also suffer from obstructive CAD and need additional antianginal treatments. Beta-blockers are not recommended for routine use in HFpEF as they do not improve outcomes, but they have a use in CAD and angina. Other antianginal treatments, such as calcium channel blockers and nitrates, have no benefit in improving heart failure outcomes. In HFmrEF and CAD, the first study on SGLT2 inhibitors in HFmrEF demonstrated that sotagliflozin reduced cardiac outcomes in patients with HF and type 2 diabetes, regardless of LVEF, with subsequent studies recommending SGLT2 inhibitors as primary treatment. Regarding other therapies, ACEI and ARBs used in HFmrEF have shown mixed results, with some beneficial effects on cardiovascular outcomes in certain subgroups. The PARAGON-HF and TOPCAT trials suggested that sacubitril–valsartan and spironolactone were effective in specific patients with HF, but their benefits remained inconclusive for certain LVEF ranges. Beta-blockers appear to have a positive impact on prognosis in HFmrEF, particularly in patients with CAD, but their role is less clear in those without CAD. In light of these studies, recent guidelines recommend using ACEI/ARB/ARNI, MRA, and beta-blocker therapy in HFmrEF with a low level of evidence (Class IIb), while ivabradine is not recommended. In HFrEF and CAD, ACE inhibitors and ARBs are essential in managing HFrEF by targeting the renin–angiotensin–aldosterone system, reducing mortality, myocardial remodeling, and hospitalizations in CAD patients. ARNI, beta-blockers, and MRAs improve hemodynamics, cardiac function, and survival, with beta-blockers controlling heart rate and MRAs reducing fluid retention and preventing cardiac remodeling. SGLT2 inhibitors, originally developed for diabetes, are now key in treating HFrEF by improving heart failure outcomes and offering renal protection. Lastly, managing angina in CAD with HFrEF requires the careful use of medications like nitrates, ranolazine, and selective calcium channel blockers, balancing symptom relief with the avoidance of worsening heart failure symptoms.

A new promising system for stratifying the prognosis of patients with HF-CAD has emerged, based on a deep pathophysiological and multisystemic evaluation. HLM (Heart Lungs Malfunction of other organs) classification assesses the severity of cardiac/lung/other-organ involvement and predicts the mortality risk at 12 months. The HLM score is an algorithm that can prove extremely useful in predicting the mortality risk of patients with HF-CAD based on the presence/absence of systolic/diastolic dysfunction of the left and right ventricles, cardiac structural damage, the presence of clinical/imaging lung involvement, and the presence/absence of malfunctions of other organs (except the heart and lungs). Apart from the parameters evaluated by HLM classification, the arrhythmic burden of patients with HF-CAD should be taken into consideration as a negative prognostic factor [121].

## 8. Future Therapies

The new pathophysiological approach to chronic coronary syndrome leaves important gaps in evidence, especially in terms of pharmacological treatment. Thus, in regard to the currently used treatment, the administration of antithrombotic therapy in patients with CCSs and ANOCA/INOCA requires complementary studies to evaluate the benefit–risk balance. Also, the recommended duration of the double antiplatelet period is unclear in the case of patients with prior revascularization, and maybe a longer period should be taken into consideration. Regarding CCS patients with HFpEF, the long-term benefit of beta-blockers is not yet established, nor is the evidence of first- and second-line antianginal therapy [122].

If we refer to more recently introduced or under-research therapies, then it is important to mention the molecules which act on the anti-inflammatory substrate of atherosclerotic disease. Therefore, the positive impact presented by the use of low doses of colchicine in patients with CCS in reducing myocardial infarction, stroke, and revascularization could set the ground for future studies in other subgroups of patients [123].

Among the immunomodulatory monoclonal antibodies, Canakinumab administered subcutaneously at 150 mg doses every 3 months reduced cardiovascular events compared to a placebo in the CANTOS study by decreasing IL-6 and hsCRP, but with the risk of increasing infectious complications by acting on a systemic level [124].

Other monoclonal antibodies, namely, Tocilizumab and Ziltivekimab, led to myocardial recovery after revascularization in patients with STEMI by reducing the plasma values of thrombotic inflammatory biomarkers, according to the recent randomized trials ASSAIL-MI and RESCUE, respectively [125,126].

Anti-inflammatory therapy appears to be a very viable line of treatment in the near future for patients with CCSs, but the potential adverse reactions and the high cost of monoclonal antibody therapies currently limit their use. Despite the remarkable discoveries that today allow us to provide tailored care to patients with CCSs, there are still several aspects to be researched and improved upon in the near future.

## 9. Conclusions

In conclusion, CAD is the most common cause of chronic HF. Despite pharmacological and interventional innovation, the incidence of chronic HF is continuously increasing, with a decreasing age of diagnosis. HF morbidity has a major impact on patients’ QoL from social, physical, and psychological points of view. The standardization and implementation of CR programs can play a crucial role in managing patients with HF and CAD, particularly in enhancing the ability to accurately measure the benefits of pharmacological treatments. These programs can help mitigate the biases associated with varying lifestyle factors, such as exercise habits, diet, and smoking, which may otherwise obscure the true effects of medications. A structured and standardized CR program ensures that all patients receive uniform guidance on lifestyle modification, physical activity, nutrition, and self-care. This uniformity helps control for external variables that could impact the progression of HF and CAD, thereby creating a more reliable baseline to evaluate pharmacological interventions. Without standardized CR, patients may adopt lifestyle changes at varying degrees, leading to inconsistent improvements that may not be directly attributable to medication alone. Moreover, through CR programs, clinicians can closely monitor patients’ adherence to lifestyle recommendations and treatment plans, enabling the collection of comprehensive data on how lifestyle factors interact with pharmacological therapies. In cases where lifestyle improvements might exaggerate the perceived benefits of a medication, a standardized CR program ensures that any observed therapeutic effects are more likely due to the medication itself, thus minimizing bias. Furthermore, integrating CR programs into routine HF and CAD management, especially those combining on-site and remote approaches, can promote consistent patient engagement and adherence, which is often challenging. These hybrid or fully online remote models make it feasible to sustain lifestyle improvements over time, improving long-term outcomes and ensuring that the benefits observed during clinical trials are more likely to be replicated in real-world settings. Thus, the management of these patients should be updated based on the most recent scientific evidence through structured patient-based programs that are economically realizable even for countries without a level of health insurance similar to Western countries.

## Figures and Tables

**Table 3:** Mechanism of action and key benefits of different drug classes in patients with CAD and concomitant HFrEF.

Drug Class	Mechanism of Action	Key Benefits in HFrEF and CAD
**ACE inhibitors/ARBs**	Blockade of renin–angiotensin–aldosterone system Reduction in afterload and preload Inhibition of myocardial remodeling	Improvement in cardiac hemodynamics Symptom relief Reduction in HF hospitalizations Reduction in mortality
**ARNIs**	Combined action of ARBs with neprilysin inhibition Enhancement of action of natriuretic peptides	Improvement in cardiac hemodynamics Reduction in HF hospitalizations Reduction in mortality
**Beta-blockers**	Blockade of sympathetic nervous system Heart rate reduction Arterial blood pressure reduction	Improvement in cardiac output Reduction in arrhythmic burden Reduction in HF hospitalizations Reduction in mortality
**MRAs**	Blockade of action of aldosterone Reduction in fluid retention Inhibition of myocardial fibrosis	Prevention of adverse cardiac remodeling Reduction in fluid overload Improvement in survival Reduction in HF hospitalizations
**SGLT2 inhibitors**	Inhibition of glucose reabsorption in proximal tubular epithelial cells of kidneys Diuresis Reduction in cardiac preload and afterload	Improvement in cardiovascular outcomes Renal protection Reduction in HF hospitalizations
**Antianginal drugs**	Vasodilation Improvement in myocardial efficiency Reduction in angina pectoris without negative inotropic effects (CCBs)	Angina pectoris relief Minimization of worsening HF symptoms

ACE—angiotensin-converting enzyme; ARB—aldosterone receptor blocker; ARNI—angiotensin receptor–neprilysin inhibitor; CAD—coronary artery disease; CCB—calcium channel blocker; HF—heart failure; HFrEF—heart failure with reduced ejection fraction; MRA—mineralocorticoid receptor agonist; SGLT2—sodium–glucose cotransporter 2.
